# Decreased expression of the *Id3* gene at 1p36.1 in ovarian adenocarcinomas

**DOI:** 10.1054/bjoc.2000.1620

**Published:** 2001-02

**Authors:** J M Arnold, S C Mok, D Purdie, G Chenevix-Trench

**Affiliations:** The Queensland Institute of Medical Research, PO Box Royal Brisbane Hospital, Herston, Queensland, 4029, Australia; Laboratory of Gynecologic Oncology, Department of Obstetrics, Gynecology and Reproduction Biology, Brigham and Women's Hospital, Harvard Medical School, Boston, MA, 02115; Department of Pathology, University of Queensland, St Lucia, Brisbane, 4067, Australia

**Keywords:** ovarian cancer, tumour suppressor gene, *Id3*, cDNA array, loss of heterozygosity

## Abstract

The molecular events that drive the initiation and progression of ovarian adenocarcinoma are not well defined. We have investigated changes in gene expression in ovarian cancer cell lines compared to an immortalized human ovarian surface epithelial cell line (HOSE) using a cDNA array. We identified 17 genes that were under-expressed and 10 genes that were over-expressed in the cell lines compared to the HOSE cells. One of the genes under-expressed in the ovarian cancer cell lines, *Id3*, a transcriptional inactivator, was selected for further investigation. *Id3* mRNA was expressed at reduced levels in 6 out of 9 ovarian cancer cell lines compared to the HOSE cells while at the protein level, all 7 ovarian cancer cell lines examined expressed the Id3 protein at greatly reduced levels. Expression of *Id3* mRNA was also examined in primary ovarian tumours and was found in only 12/38 (32%) cases. A search was conducted for mutations of *Id3* in primary ovarian cancers using single stranded conformation polymorphism (SSCP) analysis. Only one nucleotide substitution, present also in the corresponding constitutional DNA, was found in 94 ovarian tumours. Furthermore no association was found between LOH at 1p36 and lack of expression of *Id3*. These data suggest that Id3 is not the target of LOH at 1p36. © 2001 Cancer Research Campaign http://www.bjcancer.com


British Journal of Cancer (2001) 84(3), 352–359
© 2001 Cancer Research Campaign
doi: 10.1054/ bjoc.2000.1620, available online at http://www.idealibrary.com on IDEAL
http://www.bjcancer.com

### Decreased expression of the *Id3* gene at 1p36.1 in ovarian adenocarcinomas

**JM Arnold[1], SC Mok[2], D Purdie[1] and G Chenevix-Trench[1,3]**

[1]The Queensland Institute of Medical Research, PO Box Royal Brisbane Hospital, Herston, Queensland, Australia, 4029; [2]Laboratory of Gynecologic Oncology, Department of Obstetrics, Gynecology and Reproduction Biology, Brigham and Women's Hospital, Harvard Medical School, Boston, MA 02115; [3]Department of Pathology, University of Queensland, St Lucia, Brisbane, Australia, 4067

**Summary** The molecular events that drive the initiation and progression of ovarian adenocarcinoma are not well defined. We have investigated changes in gene expression in ovarian cancer cell lines compared to an immortalized human ovarian surface epithelial cell line (HOSE) using a cDNA array. We identified 17 genes that were under-expressed and 10 genes that were over-expressed in the cell lines compared to the HOSE cells. One of the genes under-expressed in the ovarian cancer cell lines, *Id3*, a transcriptional inactivator, was selected for further investigation. *Id3* mRNA was expressed at reduced levels in 6 out of 9 ovarian cancer cell lines compared to the HOSE cells while at the protein level, all 7 ovarian cancer cell lines examined expressed the Id3 protein at greatly reduced levels. Expression of *Id3* mRNA was also examined in primary ovarian tumours and was found in only 12/38 (32%) cases. A search was conducted for mutations of *Id3* in primary ovarian cancers using single stranded conformation polymorphism (SSCP) analysis. Only one nucleotide substitution, present also in the corresponding constitutional DNA, was found in 94 ovarian tumours. Furthermore no association was found between LOH at 1p36 and lack of expression of *Id3*. These data suggest that Id3 is not the target of LOH at 1p36. © 2001 Cancer Research Campaign http://www.bjcancer.com

**Keywords:** ovarian cancer; tumour suppressor gene; *Id3*; cDNA array; loss of heterozygosity

Ovarian cancer is the sixth most common cancer among women worldwide, and the leading cause of death from gynaecological neoplasias (Parkin et al, 1999). The lack of symptoms during the early stages of the disease results in the majority of women presenting with advanced tumours that do not respond well to treatment. Tumorigenesis is widely accepted as a multi-step process driven by the accumulation of molecular changes leading to alterations in the expression or function of numerous genes which may be classified as either oncogenes or tumour suppressor genes. Tumour suppressor genes have been further divided into Type I, which are usually inactivated by mutation, and Type II, which are usually inactivated by loss of expression but are not commonly mutated (Sager, 1997). The identification and characterization of these changes will lead to an improved understanding of the molecular basis of this disease and may provide opportunities for early disease detection and novel therapeutic approaches that are urgently required.

Some progress has been made in identifying genes with mutations or altered expression patterns in ovarian adenocarcinomas. For example, p53 mutations have been found in 37% of stage I and II tumours and 58% of stage III and IV tumours (Shelling et al, 1995) and the K-*ras* oncogene is activated in a proportion of these tumours, particularly those of mucinous histology (Mandai et al, 1998). There are also a number of tumour suppressor genes (e.g. *PTEN, CDKN2A, BRCA1* and *BRCA2*) in which mutations have been reported in ovarian tumours at a low frequency (Merajver et al, 1995; Foster et al, 1996; Shih et al, 1997; Obata et al, 1998).

*Received 27 April 2000*
*Revised 25 September 2000*
*Accepted 8 November 2000*

*Correspondence to:* J Arnold

It has recently been demonstrated that the expression of several putative tumour suppressor genes such as *OVCA1*, *Disabled-2*, *GPC3*, *NOEY2* and *SPARC* is lost in a proportion of ovarian adenocarcinomas (Mok et al, 1996, 1998; Bruening et al, 1999; Lin et al, 1999; Yu et al, 1999). In addition, the c-*erbB*-2, c-*fms*, and c-*myc*, oncogenes are commonly overexpressed in ovarian cancers (Yasue et al, 1987; Zhou et al, 1988; Kohler et al, 1989; Slamon et al, 1989). Despite this progress, the molecular changes associated with the development and progression of this disease remain unclear. There is increasing evidence for heterogeneity in the pathway to malignancy with respect to histologic sub-types (Mandai et al, 1998; Obata et al, 1998; Wright et al, 1999) but there is no established model for ovarian tumorigenesis.

There are a number of different methods for the comparison of global gene expression patterns between two or more tissue sources. These include differential display (Liang and Pardee, 1992), subtractive hybridization (Lee et al, 1991), serial analysis of gene expression (SAGE) (Velculescu et al, 1995), EST database comparisons (Vasmatzis et al, 1998), high-density cDNA microarrays (Schena et al, 1995), and commercially available cDNA arrays that consist of hundreds of cDNAs immobilized to nylon membranes (Peitu et al, 1996). We have investigated changes in gene expression patterns in ovarian adenocarcinoma by examining differences between an immortalized human ovarian surface epithelial cell line (HOSE) and 3 ovarian cancer cell lines using the cDNA Atlas Array available from Clontech, Inc. This method allows the rapid and simultaneous comparison of the expression of hundreds of genes in multiple cell lines. The reduced levels of expression of one gene, *Id3*, in the ovarian cancer cell lines, combined with its chromosomal localization at 1p36 (which we have shown is a common region of LOH in ovarian cancer (Imyanitov et al, 1999)), suggested that it may be important in ovarian tumorigenesis. This report presents the results of the analysis of *Id3* expression in HOSE and ovarian cancer cell lines as well as further characterization of *Id3* in primary ovarian tumours by expression and mutation analysis.

### MATERIALS AND METHODS

### Cell lines, ovarian surface epithelium cultures and primary tumours

Human ovarian surface epithelial cell lines (HOSE) 17.1 and 1.1, immortalized with a retroviral vector expressing human papillomavirus oncogenes (Tsao et al, 1995), were maintained in a medium composed of 1:1 M199:MCDB105 with 10% FCS. The HEY ovarian cancer cell line was maintained in MEMα medium with 10% FCS (Buick et al, 1985). The remainder of the ovarian cancer cell lines – OAW 42 (Wilson, 1984), OAW 28+53 (Wilson et al, 1987), PEO1 and PEO4 (Wolf et al, 1987), PEO14 (Langdon et al, 1988), JAM (Ward et al, 1987), SKOV3 (Fogh and Trempe, 1975), COLO316 (Woods et al, 1979), CAOV3 (Wong et al, 1999), OVCAR-3 (Hamilton et al, 1983), and 27/87 (derived by T Hurst) – were all maintained in RPMI 1640 with 10% FCS. Cells were harvested for RNA and protein isolation at about 80% confluence. The cell lines OAW 42, PEO1, PEO4, PEO14, JAM, SKOV3 and COLO316 were derived from serous tumours, and 27/87 from an endometrioid tumour.

Primary cultures of human ovarian surface epithelial cells were prepared according to the method of Kruk et al (1990). Epithelial cells were obtained by scraping contaminating stromal cells away from proliferating epithelial sheets and cultured in 1:1 MCDB105: Medium 199 with Earles' salts, supplemented with 20 ng $ml^{-1}$ epidermal growth factor, 400 ng $ml^{-1}$ hydrocortisone and 15% fetal calf serum. The distinctive cellular morphology was used to certify that the cultures were epithelial cells. In one case, RNA was extracted directly from the peeled epithelial cells without culturing.

DNA was obtained from 95 patients with ovarian neoplasms undergoing surgery. There were 67 serous tumours, 12 endometrioid tumours, 9 mucinous tumours, 5 clear-cell tumours, and 2 tumours of mixed histology. The series included 1 benign and 10 low-malignant potential (LMP) tumours as well as 84 malignant tumours. All patients were staged at laparotomy, in accordance with the recommendations of the International Federation of Gynaecology and Obstetrics (FIGO). Of the LMP tumours, 5 were FIGO stage 1 and 4 were stage 3 (one was of unknown stage) and of the malignant tumours, 7 were stage 1, 4 stage 2, 65 stage 3 and 8 stage 4. Constitutional DNA from peripheral blood was available for all cases. RNA was available from a subset of 38 tumours.

### Isolation of DNA and RNA

Tumour tissue was dissected free from necrotic and connective tissue, and mechanically dispersed prior to collagenase treatment (0.1 mg $ml^{-1}$ in Hanks balanced salt solution). Dead and red cells were then removed by ficoll-paque, and genomic DNA was extracted by the salting-out method as described elsewhere (Chenevix-Trench et al, 1992). The purity of the resulting DNA is supported by the high frequency of LOH on chromosome 17 detected in ovarian tumour DNA prepared by this method (Leary et al, 1995). Total RNA was extracted from primary tumours or sub-confluent cultured cell lines using the Tri-reagent (Sigma), following the manufacturers' instructions. PolyA+ RNA was prepared from this total RNA using Dynabeads (Dynal) according to the recommended protocol.

### Hybridization to array membrane

Hybridizations to the Atlas Array (Clontech, Inc.) were performed strictly as recommended by the manufacturer. Probes were prepared by reverse transcription from 1 μg of polyA+ RNA and hybridized to the membrane overnight in ExpressHyb (Clontech, Inc.) solution at 68˚C, followed by stringent washing and autoradiography. The names of all genes and controls on the array, and their accession numbers, can be obtained from http://www.clontech.com/clontech/APR97UPD/Atlaslist.html.

### Semi-quantitative RT-PCR

RT-PCR was performed on 1 μg of total RNA in a total volume of 20 μl incorporating $^{33}$P-dATP using standard PCR cycling conditions. Primer pairs were designed to amplify 300–500 bp cDNA fragments and spanned at least 1 intron to ensure quantitation was assessed only on amplified cDNA. Multiplex PCR was carried out incorporating $^{33}$P-ATP using primers for β-actin as an internal control. The reaction was stopped and 5 μl of product removed 3 times between cycles 16 and 30 to ensure linear amplification and the products were run on a denaturing acrylamide gel prior to autoradiography. The primer sequences are Id3 (5′-CTCCG-GAACTTGTCATCTCCAACG and 5′-GTTCATAAATCAGGGC-AACAGAACA), β-actin (5′-CGTGACAAT AAGGAGAAGCTG-TGC and 5′-CTCAGGAGGAGCAATGATCTTGAT) and GAPDH (5′-ATGGATCCAGTCCATGCCATCACTGCC and 5′ATGGTAC-CGAGGTCCACCACCCTGTTG).

### Northern blot analysis

RNA was denatured and electrophoresed on a formaldehyde-agarose gel and transferred to a nylon membrane (Amersham Hybond N+) by capillary blotting overnight according to standard protocols (Sambrook et al, 1989). The RNA was then fixed to the membrane by UV irradiation. Probes were prepared from RT-PCR products by random priming (Amersham Megaprime) and hybridization was carried out for 2 hours in ExpressHyb solution (Clontech, Inc.) at 65˚C (for random primed probes) before a standard washing procedure.

### Protein extraction and Western blotting

Harvested cells were lysed by freeze-thawing in 25 mM HEPES, pH 8, 0.25 M sucrose, 1 mM EGTA, 5 mM $MgSO_4$, 50 mM NaF, 1 mM DTT and protease inhibitors (Complete™ protease inhibitors cocktail, Boehringer Mannheim), followed by mild sonication. Cell debris was removed by centrifugation and protein concentrations determined using the BioRad Protein Assay reagent. Proteins and prestained SDS-PAGE standards (Biorad) were electrophoresed on a 15% SDS-PAGE and then electroblotted onto Hybond-C membrane (Amersham). The membrane was washed in 0.1% Tween 20 (PBT) and blocked with 5% skim milk powder before incubating with 0.01 μ$ml^{-1}$ affinity purified, rabbit polyclonal anti-Id3 antibody (Santa Cruz) or 1:200 anti β-actin antibody (Sigma) overnight. The membrane was then

© 2001 Cancer Research Campaign incubated with the appropriate HRP-conjugated secondary antibody for 2 hours before washing, followed by enzyme chemiluminescence and autoradiography.

### Loss of heterozygosity (LOH) analyses

LOH was analysed at 1p36 with the D1S2734 marker. PCR amplification was carried out for 35 cycles in the presence of $^{33}$P-dATP and PCR products were analysed on a denaturing polyacrylamide gel. LOH was scored conservatively as a substantial reduction in the intensity of one allele by two independent observers one of whom was blind with respect to sample identity.

### Single strand conformation polymorphism analysis

Primers were designed to amplify the coding exons of the human *Id3*. The first exon of 351 bp required two overlapping amplimers (260 bp [5′-CAGGCAGGCTCTATAAGTGACC and 5′-GTAGCAGTGGTTCATGTCGTCC] and 280 bp [5′-GTCG-GAACGCAGTCTGGCCATCG and 5′-CAGCCCTGTCCCGA CTTCGAGG]) while the second 85 bp exon was amplified in a single fragment (266 bp [5′-CTTCCCATCCAGGTAAGCCTCG and 5′-CGACTGCCAACTCCAGGACTTGC]). The third exon of 448 bp encoded only the 3′UTR and was not screened for mutations. 94 of the available 95 primary ovarian tumours were screened and constitutional DNA was available from blood in all cases. DNA samples were amplified in the presence of $^{33}$P-dATP using standard PCR cycling conditions with annealing at 60˚C, denatured at 95˚C for 5 minutes and then electrophoresed on 0.5 × MDE (FMC Biotech) gel overnight at room temperature. Samples with aberrantly migrating bands were re-amplified and sequenced directly using ABI Prism Big Dye terminator cycle sequencing ready reagent kit (PE Applied Biosystems) and analysed on an ABI 377 sequencer.

### Statistical analyses

Survival curves, survival probabilities and estimated mean survival times were calculated according to the Kaplan-Meier method. The differences between the survival curves for the Id3-positive and Id3-negative groups were evaluated using the log rank test. Fisher's Exact test was used to evaluate the association between *Id3* expression and LOH at 1p36 and the test for trend was used to examine association between *Id3* expression and stage or grade.

### RESULTS

A cDNA array containing 588 known human cDNA fragments fixed to a nylon membrane was used to analyse differences in gene expression between normal OSE cells and ovarian tumour cell lines. This array contains cDNAs coding for proteins of many different functional groups including oncogenes and tumour suppressor genes. Gene expression in the immortalized OSE line, HOSE 17.1, was first compared to itself in 2 separate hybridizations to confirm reproducibility and then to that in 3 arbitrarily chosen ovarian tumour cell lines derived from serous tumours (OAW42, PEO1 and JAM).

A visual comparison was made between the intensities of the signals from the array genes in each cell line using signal intensities from housekeeping genes as an internal control. This identified 17 genes (AXL/UFO tyrosine kinase receptor, p16$^{INK4}$, integrin linked kinase, Id3, cAMP dependent transcription factor ATF4, p21$^{WAF1/CIP1}$, TFIID subunit TAF1131, intercellular adhesion molecule-1, neural cadherin, CD44 antigen, fibronectin receptor alpha subunit, fibronectin receptor beta subunit, bone morphogenetic protein-1, monocyte chemotactic protein-1, vascular endothelial growth factor-related protein, heparin-binding growth factor-1 and follistatin-related protein) that were expressed at higher levels in HOSE 17.1 compared with the ovarian cancer cell lines. Another 10 genes (v-ERBA-related protein EAR-1, v-ERBA-related protein EAR-2, RAF proto oncogene, galactosyltransferase-associated protein kinase, protein kinase CLK, NADPH cytochrome p450 reductase, transcription factor ETR103, transcription factor ETR101, integrin alpha 1 and integrin alpha 7B) were expressed at higher levels in all 3 ovarian cancer cell lines, compared with the HOSE 17.1 cell line.

From the 17 putative tumour suppressor genes, the 6 with the greatest differences in levels of expression but not previously linked to ovarian cancer (cAMP-dependent transcription factor ATF-4, integrin linked kinase, neural cadherin, intercellular adhesion molecule-1, monocyte chemotactic protein-1 and Id3) were selected for further analysis by semi-quantitative RT-PCR in HOSE cell lines, short-term cultures of OSEs and ovarian cancer cell lines (data not shown). There was no evidence for decreased expression in the ovarian cancer cell lines compared to the OSE cells for the cAMP-dependent transcription factor ATF-4, integrin linked kinase or neural cadherin. In contrast, intercellular adhesion molecule-1, monocyte chemotactic protein-1 and Id3 were expressed by all the ovarian surface epithelial cell lines but by only 2/6, 1/4 or 2/5 of the ovarian cancer cell lines tested, respectively. Id3 has been mapped to chromosome 1, at band 1p36.1 (Deed et al, 1994), a common region of loss of heterozygosity (LOH) in a wide variety of solid human cancers (Ragnarsson et al, 1999). Previous studies in our laboratory have found 43% LOH at 1p36 in ovarian adenocarcinomas (Imyanitov et al, 1999). On the basis of this LOH data, and the loss of expression of *Id3* in most of the ovarian cancer cell lines examined, we selected Id3 for further study of its expression and mutation profile in ovarian adenocarcinomas.

Due to the possible influence of culture conditions on the level of *Id3* RNA expression, RT-PCR was first carried out to compare expression in peeled (uncultured) OSE cells to HOSE 17.1 and

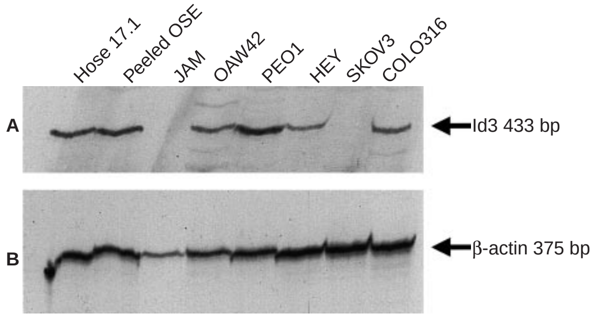

**Figure 1** RT-PCR analysis of Id3 expression in peeled (uncultured) OSE cells, immortalized HOSE cells and ovarian cancer cell lines. RT-PCR was carried out in a multiplex reaction with β-actin as an internal control on peeled OSE cells, HOSE 17.1 and ovarian cancer cell lines. PCR products were removed for analysis at 20, 25 and 30 cycles. The *Id3* products after 30 cycles and β-*actin* products after 20 cycles are shown here

 © 2001 Cancer Research Campaign ovarian cancer cell lines (Figure 1). This revealed that the level of *Id3* mRNA expression was similar in the peeled OSE cells to HOSE 17.1. The HOSE 17.1 cells were therefore used as a control cell population for the remainder of this investigation due to the limited availability of peeled OSE cells. *Id3* mRNA expression was not detected by RT-PCR in 2 out of 6 ovarian cancer cell lines (JAM and SKOV3), while the other 4 cell lines (OAW42, PEO1, HEY and COLO316) had similar levels to the HOSE 17.1 cells.

*Id3* mRNA expression was further analysed in the cell lines by Northern blot analysis. Expression was observed in both of the HOSE cell lines, although the level of expression was lower in HOSE 1.1 than HOSE 17.1 (Figure 2). Similar amounts of *Id3* mRNA to those in HOSE 17.1 were observed in 3 of the ovarian cancer cell lines (OAW 42, COLO316 and CAOV3) but significantly weaker expression was found in the other 6 cell lines examined. Western blotting detected a protein of 15 kDa corresponding to Id3, at similar levels in both of the HOSE cell lines (Figure 3). However, expression was much lower in 5 of the ovarian cancer cell lines and almost undetectable in the COLO316 and PEO14 cell lines.

Having established that the expression of *Id3* is lower that the HOSE cell lines at both the mRNA and protein levels in most of the ovarian cancer cell lines, we next investigated its expression in primary ovarian tumours. Expression was analysed on a series of 37 primary ovarian adenocarcinomas using semi- quantitative RT-PCR with β-actin as an internal control (Figure 4). No expression at all was detected in 26/37 (70%) cases after 30 cycles of amplification. The remaining cases had variable levels of expression, but in most cases appeared to express less *Id3* message than the HOSE cells. This finding was supported by Northern blot analysis which showed expression in the HOSE cells but in only 1/10 primary tumours examined (Figure 5). RT-PCR was not performed on this single tumour showing expression by Northern analysis but a combination of RT-PCR and Northern data showed that *Id3* expression was not detected in 26/38 (68%) primary tumours.

To investigate the molecular basis of the down-regulation of *Id3* in primary ovarian adenocarcinomas, the *Id3* gene was analysed for mutations in a series of 94 primary ovarian tumours using single strand conformation polymorphism (SSCP) analysis. This included 37 of the tumours for which we had *Id3* expression data. There was only one nucleotide change detected in the entire series, which was a G to A conversion resulting in the change of codon 105 from GCT (alanine) to ACT (threonine) (Table 1). Both the tumour and the corresponding constitutional DNA were heterozygous for this change. LOH at 1p36 was assessed with the D1S2734 microsatellite marker in the cases for which *Id3* expression had been analysed. LOH was detected in 9/30 (30%) informative cases.

Analysis of the survival data for the patients whose tumours expressed *Id3*, compared to those whose tumours did not, revealed a weak relationship between *Id3* expression and improved survival, although this was not significant ($P$ = 0.43, log rank test). No correlation was found between lack of *Id3* expression and LOH at 1p36 ($P$ = 0.68, Fisher's Exact test), nor was there any trend towards loss of expression with higher stage ($P$ = 1.0, Test for Trend) or grade ($P$ = 0.68, Test for Trend).

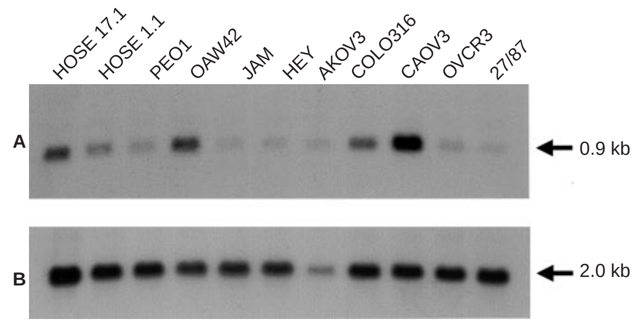

**Figure 2** Northern blot analysis of Id3 expression in HOSE and ovarian cancer cell lines. Each lane contains 2 μg of polyA+ RNA. (**A**) Hybridization with the *Id3* probe, (**B**) hybridization with the β-*actin* probe

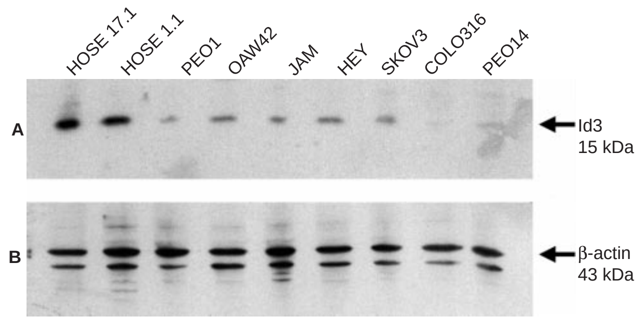

**Figure 3** Western blot analysis of Id3 expression in HOSE and ovarian cancer cell lines. Each lane contains 20 μg of total cellular protein. (**A**) Reactivity with the anti-Id3 antibody; (**B**) reactivity with the anti-β-actin antibody

### DISCUSSION

We have used cDNA array analysis to investigate differences between human ovarian surface epithelial (HOSE) cell lines and 3 serous ovarian cancer cell lines in the expression of a panel of 588 known human genes. A total of 17 genes were identified with reduced expression and another 10 genes with higher expression in the ovarian cancer cell lines compared to the HOSE cell line. Wang et al (1999) and Schummer (1999) also used cDNA microarrays to monitor gene expression changes in ovarian tumours compared to normal ovaries. There were no common genes between those identified in our study and those in the top 15 over-expressed or under-expressed known genes reported by Wang et al (1999) or the 43 known genes found to be over-expressed by Schummer et al (1999). This may be a reflection of the different cDNAs included in the respective arrays.

Many of the genes indicated in this study as being over- or under-expressed in the ovarian cancer cell lines are well-studied tumour suppressor genes (eg $p16^{INK4}$ and $p21^{WAF1/CIP1}$) or oncogenes (e.g. c-*raf* ). Several of the other genes shown to be differentially expressed have been reported as having a role in the control of cell growth or transformation, such as the AXL/UFO tyrosine kinase receptor (Janssen et al, 1991), the fibronectin receptor (reviewed in Ruoslahti 1996), the cAMP-dependent transcription-factor, ATF-4 (Mielnicki et al, 1996) and the galactosyltransferase-associated protein kinase (Li et al, 1995). Therefore, on the basis of the reported functions of the genes that are apparently

*© 2001 Cancer Research Campaign* 

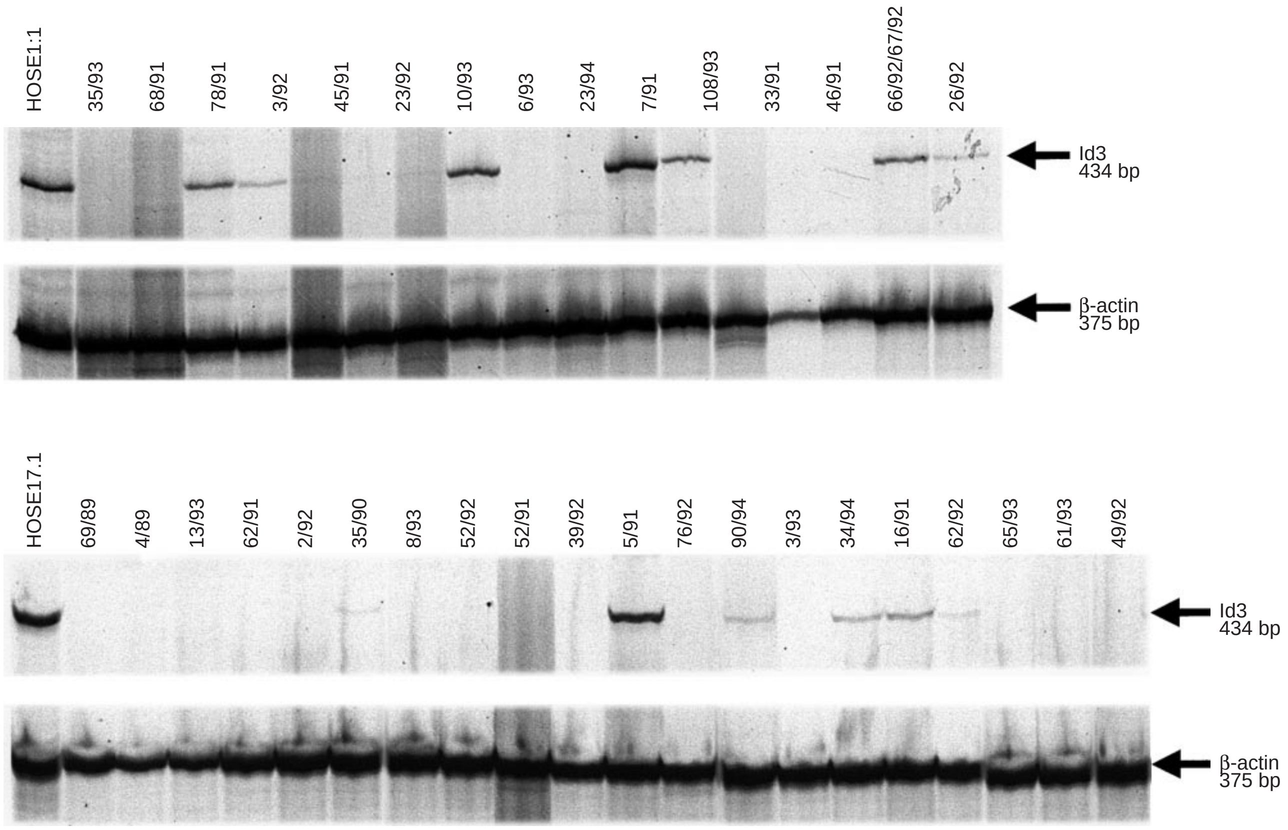

**Figure 4** RT-PCR analysis of Id3 expression in ovarian primary tumours. RT-PCR was carried out in a multiplex reaction with β-actin as an internal control for 30 cycles on HOSE 17.1 cells and 37 primary ovarian cancers

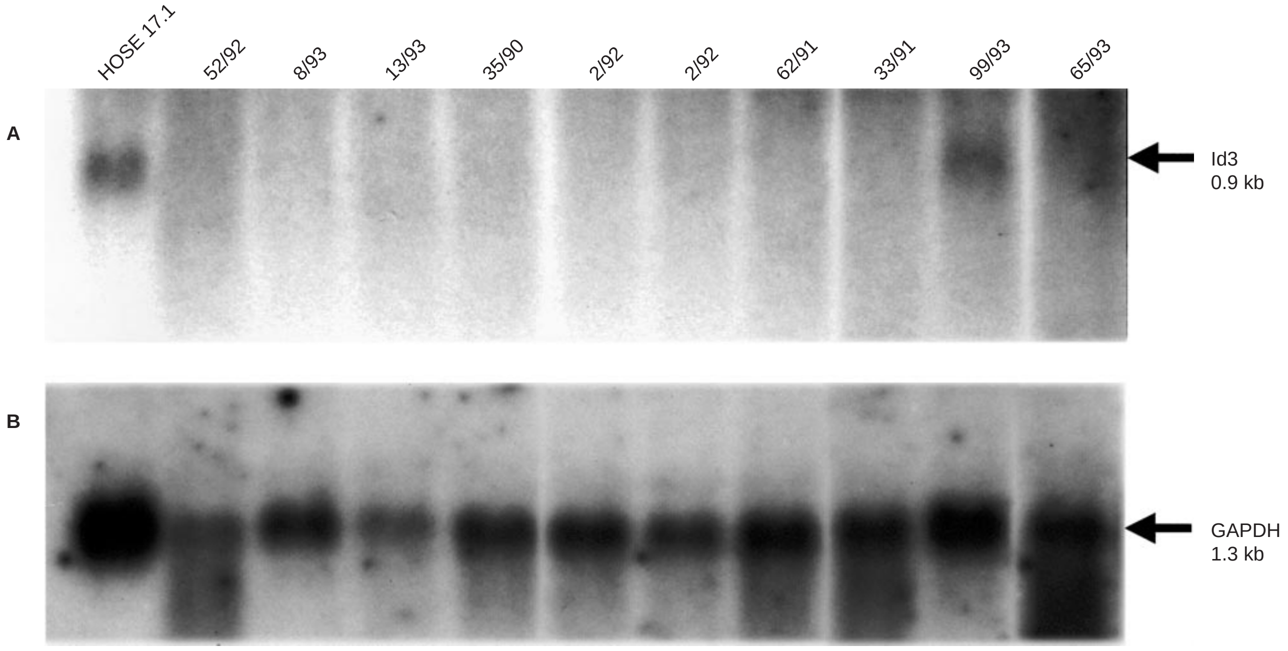

**Figure 5** Northern blot analysis of Id3 expression in ovarian primary tumours. Each lane contains 5 μg of total RNA. (**A**) Hybridization with the *Id3* probe; (**B**) hybridization with *GAPDH* probe.

© 2001 Cancer Research Campaign

**Table 1** Id3 expression, 1p36 LOH status, SSCP results and clinicopathological data for 38 primary ovarian cancers

| | | | | Expression | | LOH 1p36 | |
|---|---|---|---|---|---|---|---|
| **Case** | **Histo** | **Stage** | **Grade** | **Nthrn** | **RT PCR** | **D1S2734** | **SSCP shift** |
| 69/89 | SER | 3 | 2 | – | – | NL | no |
| 35/90 | SER | 3B | 2/3 | – | – | L | no |
| 5/91 | SER | 3 | 2 | | XXX | Nl | no |
| 7/91 | SER | 3 | 2 | | XXX | L | no |
| 16/91 | SER | 2 | 3 | | X | NL | no |
| 33/91 | ENDO | 4 | 3 | – | – | NL | no |
| 45/91 | SER | 3C | NA | | – | NL | no |
| 46/91 | ENDO | 3B | 2 | | – | NL | no |
| 52/91 | SER | 2B | 2 | | – | NL | no |
| 62/91 | SER | 3 | 3 | – | – | | no |
| 68/91 | SER | 3 | 3 | | – | NL | no |
| 78/91 | SER | 3 | 2/3 | | XX | NL | no |
| 2/92 | SER | 3 | 2 | – | – | NL | no |
| 3/92 | SER | 3 | 3 | | X | L | no |
| 4/92 | SER | 3B | 3 | | – | L | no |
| 23/92 | SER | 3 | 3 | | – | NL | not tested |
| 26/92 | CCC | 4 | 2 | | X | NL | no |
| 39/92 | SER | 3 | 2 | | – | NL | no |
| 49/92 | CCC | 3B | NA | | – | | no |
| 52/92 | SER | 3 | 2 | – | – | L | no |
| 62/92 | SER | 3C | 2/3 | | – | NL | no |
| 66/92 | ENDO | 3C | 3 | | – | NS | no |
| 67/92 | ENDO | 1C | 2/3 | | XX | NL | no |
| 76/92 | SER | 3 | 2/3 | | – | NL | no |
| 3/93 | SER | 3C | 3 | | – | NL | no |
| 6/93 | SER | 3C | 2/3 | | XX | NL | no |
| 8/93 | SER | 3C | 2/3 | – | – | Nl | no |
| 10/93 | SER | 3C | 2/3 | | – | NS | no |
| 13/93 | SER | 3C | NA | – | – | L | no |
| 35/93 | SER | 3C | 3 | | – | L | G to A[a] |
| 61/93 | ENDO | 4 | 2 | | – | NL | no |
| 65/93 | ENDO | 3 | 2/3 | – | – | L | no |
| 99/93 | ENDO | 4 | 3 | XX | | NL | no |
| 108/93 | SER | 3C | 2 | | XX | NL | no |
| 23/94 | SER | 3 | 3 | | – | | no |
| 25/94 | SER | 3C | 2 | | – | | no |
| 34/94 | SER | 3C | 3 | | X | L | no |
| 90/94 | SER | 4 | 2 | | X | NL | no |

[a]polymorphism = present also in constitutional DNA. NA = not available; – = no expression detected; x, xx and xxx = increasing levels of expression; NL = no loss; NI = not informative; L = loss; NS = not scored as alleles too close together.

differentially expressed in the ovarian cancer cell lines compared to the HOSE, most appear to be plausible candidates for a role in ovarian cancer.

The Id (inhibitor of differentiation/DNA binding) proteins belong to a class of transcription factors known as the helix-loop-helix (HLH) proteins as they contain an amino acid sequence predicted to form a helix-loop-helix structure which mediates the dimerization of these proteins (Murre et al, 1989). A sub-group of HLH proteins, termed the basic HLH proteins (bHLH), contain a domain of basic amino acids amino-terminal to the HLH domain which mediates the binding of these protein dimers to a DNA sequence element called the E-box (CANNTG), leading to $G_1$ cell cycle arrest and the transcription of differentiation-specific genes (Murre et al, 1989; Blackwell and Weintraub, 1990). The four Id proteins lack the basic DNA-binding domain and their heterodimerization with other bHLH transcription factors inhibits DNA-binding and inactivates transcription in a dominant negative manner, thus inhibiting lineage-specific gene expression (reviewed in Norton et al, 1998; Israel et al, 1999).

An accumulating body of expression and functional evidence suggests that the Id proteins may function as oncogenes. In addition to inhibiting $G_1$ cell cycle arrest and differentiation, *Id* genes have been shown to enhance cell cycle progression (reviewed in Norton et al, 1998; Israel et al, 1999). Over-expression of *Id* genes can induce apoptosis in serum-deprived fibroblast cells and co-transfection of *Id3* with the anti-apoptotic Bc12 or $Bc1X_L$ genes results in immortalization of primary rat embryo fibroblasts (Norton and Atherton, 1998). Furthermore, *Id* gene expression has been reported in human cell lines derived from a variety of different tumours (Israel et al, 1999) and Id proteins are required for vascularization of tumours (Lyden et al, 1999).

Despite this putative oncogenic role, Northern and Western blot data presented here indicate that the expression of Id3 is reduced in most ovarian cancer cell lines relative to uncultured or immortalized OSE cells. Furthermore expression of Id3 was not detected at the mRNA level in 26/38 (68%) of primary tumours. However, there was no association between lack of expression and LOH on 1p36, nor did we find any mutations in the *Id3* gene. These data suggest that *Id3* is not the target of LOH at 1p36. Whether it functions as a type II tumour suppressor gene, down-regulated in ovarian tumours, is yet to be determined.

*© 2001 Cancer Research Campaign* 

### ACKNOWLEDGEMENTS

We thank T Hurst for culturing the OSE cells. This work was supported by the Queensland Cancer Fund and National Health and Medical Research Council of Australia.

### REFERENCES

Blackwell TK and Weintraub H (1990) Differences and similarities in DNA-binding preferences of MyoD and E2A protein complexes revealed by binding site selection. *Science* **250**: 1104–1110

Bruening W, Prowse AH, Schultz DC, Holgado-Madruga M, Wong A and Godwin AK (1999) Expression of OVCA1, a candidate tumour suppressor, is reduced in tumours and inhibits growth of ovarian cancer cells. *Cancer Res* **59**: 4973–4983

Buick RN, Pullano R and Trent JM (1985) Comparative proterties of five human ovarian adenocarcinoma cell lines. *Cancer Res* **45**: 3668–3676

Chenevix-Trench G, Leary J, Kerr J, Michel J, Kefford R, Hurst T, Parsons P, Friedlander M and Khoo SK (1992) Frequent loss of heterozygosity on chromosome 18 in ovarian adenocarcinoma which does not always include the DCC locus. *Oncogene* **7**: 1059–1065

Deed RW, Hirose T, Mitchell ELD, Santibanez-Koref MF and Norton JD (1994) Structural organisation and chromosomal mapping of the human *Id-3* gene. *Gene*, **151**: 309–314

Fogh J and Trempe G (1975) New human cell lines. In: Fogh J (ed) *Human tumour cells in vitro*, pp 155–159., Plenum: New York

Foster KA, Harrington P, Kerr J, Russell P, DiCoccio RA, Scott IV, Jacobs I, Chenevix-Trench G, Ponder BAJ and Gayther SA (1996) Somatic and germline mutations of the BRCA2 gene in sporadic ovarian cancer. *Cancer Res* **56**: 3622–3625

Hamilton TC, Young RC, McKoy WM, Grotzinger KR, Green JA, Chu EW, Whang-Peng J, Rogan AM, Green WR and Ozols RF (1983) Characterization of a human ovarian carcinoma cell line (NIH:OVCAR-3) with androgen and estrogen receptors. *Cancer Res* **43**: 5379–5389

Imyanitov EN, Birrell GW, Filippovich I, Sorokina N, Arnold J, Mould M, Wright K, Walsh M, Mok SC, Lavin MF, Chenevix-Trench G and Khanna KK (1999) Frequent loss of heterozygosity at 1p36 in ovarian adenocarcinoma but the gene encoding p73 is unlikely to be the target, *Oncogene* **18**: 4640–4642

Israel MA, Hernandez M-C, Florio M, Andres-Barquin PJ, Mantani A, Carter JH and Julin CM (1999) *Id* gene expression as a key mediator of tumour cell biology. *Cancer Res* **59**: 1726s–1730s

Janssen JW, Schulz AS, Steenvoorden AC, Schmidberger M, Strehl S, Ambros PF and Bartram CR (1991) A novel putative tyrosine kinase receptor with oncogenic potential. *Oncogene* **6**: 2113–2120

Kohler M, Janz I, Wintzer HO, Wagner E and Bauknecht T (1989) The expression of EGF receptors, EGF-like factors and c-myc in ovarian and cervical carcinomas and their potential clinical significance. *Anticancer Res* **9**: 1537–1548

Kruk PA, Maines-Bandiera SL and Auersperg NA (1990) Simplified method to culture ovarian surface epithelium. *Laboratory Investigation* **63**: 132–136

Langdon SP, Lawrie SS, Hay FG, Hawkes MM, McDonald A, Hayward IP, School DJ, Hilgers J, Leonard RCF and Smyth JF (1988) Characterization and properties of nine human ovarian adenocarcinoma cell lines. *Cancer Res* **48**: 6166–6172

Leary JA, Kerr J, Chenevix-Trench G, Doris CP, Hurst T, Houghton CRS and Friedlander ML (1995) Increased expression of the NME1 gene is associated with metastasis in epithelial ovarian cancer. *Int J Cancer* **64**: 189–195

Lee SW, Tomasetto C and Sager R (1991) Positive selection of tumour suppressor genes by subtractive hybridisation. *Proc Natl Acad Sci USA* **88**: 2825–2829

Li S, MacLachlan TK, De Luca A, Claudio PP, Condorelli G and Giordano A (1995) The cdc-2-related kinase, PISSLRE, is essential for cell growth and acts in G2 phase of the cell cycle. *Cancer Res* **55**: 3992–3995

Liang P and Pardee AB (1992) Differential display of eukaryotic messenger RNA by means of the polymerase chain reaction. *Science* **257**: 967–971

Lin H, Huber R, Schlessinger D and Morin PJ (1999) Frequent silencing of the GPC3 gene in ovarian cancer cell lines. *Cancer Res* **59**: 807–810

Lyden D, Young AZ, Zagzag D, Yan W, Gerald W, O'Reilly R, Bader RO, Zhuang A, Manova K and Benezra R (1999) Id1 and Id3 are required for neurogenesis, angiogenesis and vascularization of tumour xenografts. *Nature* **401**: 670–677

Mandai M, Konishi I, Kuroda H, Komatsu T, Yamamoto S, Nanbu K, Matsushita K, Fukumoto M, Yamabe H and Mori T (1998) Heterogeneous distribution of K-ras-mutated epithelia in mucinous ovarian tumours with special reference to histopathology. *Hum Pathol* **29**: 34–40

Merajver SD, Pham TM, Caduff RF, Chen M, Poy EL, Cooney KA, Weber BL, Collins FS, Johnston C and Frank TS (1995) Somatic mutations in the BRCA1 gene in sporadic ovarian tumours. *Nature Genetics* **9**: 439–443

Mielnicki LM, Hughes RG, Chevray PM and Pruitt SC (1996) Mutated ATF-4 suppresses c-Ha-ras oncogene transcript levels and cellular transformation in NIH3T3 fibroblasts. *Biochem Biophys Res Comm* **228**: 586–595

Mok SC, Chan WY, Wong KK, Muto MG and Berkowitz RS (1996) SPARC, an extracellular matrix protein with tumour-suppressing activity in human ovarian epithelial cells. *Oncogene* **12**: 1895–1901

Mok SC, Chan WY, Wong KK, Cheung KK, Lau CC, Ng SW, Baldini A, Colitti CV, Rock CO and Berkowitz RS (1998) DOC-2, a candidate tumour suppressor gene in human epithelial ovarian cancer. *Oncogene* **16**: 2381–2387

Murre C, McCaw PS and Baltimore D (1989) A new DNA binding and dimerization motif in immunoglobulin enhancer binding, daughterless, in MyoD, and myc proteins. *Cell* **56**: 777–783

Norton JD and Atherton GT (1998) Coupling of cell growth control and apoptosis functions of Id proteins. *Mol Cell Biol* **18**: 2371–2381

Norton JD, Deed RW, Craggs G and Sablitzky F (1998) Id helix-loop-helix proteins in cell growth and differentiation. *Trends Cell Biol* **8**: 58–65

Obata K, Morland SJ, Watson RH, Hitchcock A, Chenevix-Trench G, Thomas EJ and Campbell IG (1998) Frequent PTEN/MMAC mutations in endometrioid but not serous or mucinous epithelial ovarian tumours. *Cancer Res* **58**: 2095–2097

O'Bryan JP, Frye RA, Cogswell PC, Neubauer A, Kitch B, Prokop C, Espinosa R, Le Beau MM, Earp HS and Liu ET (1991) ax1, a transforming gene isolated from primary human myeloid leukemia cells, encodes a novel receptor tyrosine kinase. *Mol Cell Biol* **11**: 5016–5031

Parkin DM, Pisani P and Ferlay J (1999) Estimates of the worldwide incidence of 25 major cancers in 1990. *Int J Cancer* **80**: 827–841

Peitu G, Alibert O, Guichard V, Lanny B, Bois F, Leroy E, Mariage-Samson R, Houlgatte R, Soularue P and Auffray C (1996) Novel gene transcripts preferentially expressed in human muscles revealed by quantitative hybridization of a high density cDNA array. *Genome Res* **6**: 492–503

Ragnarrson G, Eiriksdottir G, Johannsdottir JT, Jonasson JG, Egilsson V and Ingvarsson S (1999) Loss of heterozygosity at chromosome 1p in different solid human tumours: association with survival. *Br J Cancer* **79**: 1468–1474

Ruoslahti E (1996) Integrin signalling and matrix assembly. *Tumour Biol* **17**: 117–124

Sager R (1997) Expression genetics in cancer: shifting the focus from DNA to RNA. *Proc Natl Acad Sci USA* **94**: 952–955

Sambrook J, Fritch EF and Maniatis T (1989) *Molecular cloning: A laboratory manual, second edition*. Cold Spring Harbor Laboratory Press: New York

Schena M, Shalon D, Davis RW and Brown PO (1995) Quantitative monitoring of gene expression patterns with a complementary DNA microarray. *Science* **270**: 467–470

Schummer M, Ng WV, Bumgarner RE, Nelson PS, Schummer B, Bednarski DW, Hassel L, Baldwin RL, Karlan BY and Hood L (1999) Comparative hybridisation of an array of 21 500 ovarian cDNAs for the discovery of genes overexpressed in ovarian carcinomas. *Gene* **238**: 375–385

Shelling AN, Cooke IE and Ganesan TS (1995) The genetic analysis of ovarian cancer. *Br J Cancer* **72**: 521–527

Shih Y, Kerr J, Liu J, Hurst T, Khoo S, Ward B, Wainwright B and Chenevix-Trench G (1997) Rare mutations and no hypermethylation at the CDKN2A locus in epithelial ovarian tumours. *Int J Cancer* **70**: 508–511

Slamon DJ, Godolphin W, Jones LA, Holt JA, Wong SG, Keith DE, Levin WJ, Stuart SG, Udove J, Ullrich A and Press MF (1989) Studies of the HER-2/neu proto-oncogene in human breast and ovarian cancer. *Science* **244**: 707–712

Tsao S-W, Mok SC, Fey EG, Fletcher JA, Wan TSK, Chew E-C, Muto MG, Knapp RC and Berkowitz RS (1995) Characterization of human ovarian surface epithelial cells immortalized by human papilloma viral oncogenes (HPV-E6E7 ORFs). *Exp Cell Res* **218**: 499–507

Vasmatzis G, Essand M, Brinkmann U, Lee B and Pastan L (1998) Discovery of three genes specifically expressed in the human prostate by expressed sequence tag database analysis. *Proc Natl Acad Sci USA* **95**: 300–304

Velculescu VE, Zhang L, Vogelstein B and Kinzler KW (1995) Serial analysis of gene expression. *Science* **270**: 484–487

Wang K, Gan L, Jeffery E, Gayle M, Gown AM, Skelly M, Nelson PS Ng WV, Schummer M, Hood L and Mulligan J (1999) Monitoring gene expression profile changes in ovarian carcinomas using cDNA microarray. *Gene* **229**: 101–108

Ward BG, Wallace K, Shepherd JH and Balkwill FR (1987) Intraperitoneal xenografts of human epithelial ovarian cancer in nude mice. *Cancer Res* **47**: 2662–2667

Wilson AP (1984) Characterization of a cell line derived from the ascites of a patient with papillary serious cystadenocarcinoma of the ovary. *J Natl Cancer Inst* **72**: 513–520

© 2001 Cancer Research Campaign

Wilson AP, Ford CHJ, Newman CE and Howell A (1987) *Cis*-platinum and ovarian carcinoma: *in vitro* chemosensitivity of cultured tumour cells from patients receiving high dose *cis*-platinum as first line treatment. *Br J Cancer* **56**: 763–773

Wolf CR, Hayward IP, Lawrie SS, Buckton K, McIntyre MA, Adams DJ, Lewis AD, Scott ARR and Smyth JF (1987) Cellular heterogeneity and drug resistance in two ovarian adenocarcinoma cell lines derived from a single patient. *Int J Cancer* **39**: 695–702

Wong AST, Maines-Bandiear SL, Rosen B, Wheelock MJ, Johnson KR, Leung PCK, Roskelley CD and Auersperg N (1999) Constitutive and conditional cadherin expression in cultured human ovarian surface epithelium: influence of family history on ovarian cancer. *Int J Cancer* **81**: 180–188

Woods LK, Morgan RT, Quinn LA, Moore GE, Semple TU and Stedman KE (1979) Comparison of four new cell lines from patients with adenocarcinoma of the ovary. *Cancer Res* **39**: 4449–4459

Wright K, Wilson P, Morland S, Campbell I, Walsh M, Hurst T, Ward B, Cummings M and Chenevix-Trench G (1999) Beta-catenin mutation and expression analysis in ovarian cancer: exon 3 mutations and nuclear translocation in 16% of endometrioid tumours. *Int J Cancer* **82**: 625–629

Yasue H, Takeda A and Ishibashi M (1987) Amplification of the c-myc gene and the elevation of its transcripts in human ovarian tumour lines. *Cell Struct Funct* **12**: 121–126

Yu Y, Xu F, Peng H, Fang X, Zhao S, Li Y, Cuevas B, Kuo W-L, Gray JW, Siciliano M, Mills GB and Blast Jr RC (1999) NOEY2 (ARH1), an imprinted putative tumour supressor gene in ovarian and breast carcinomas. *Proc Natl Acad Sci USA* **96**: 412–219

Zhou DJ, Gonzalez-Cadavid N, Ahuja H, Battifora H, Moore GE and Cline MJ (1988) A unique pattern of proto-oncogene abnormalities in ovarian adenocarcinomas. *Cancer* **62**: 1573–1579

*© 2001 Cancer Research Campaign*
